# In Vitro Activity of Carbosilane Cationic Dendritic Molecules on Prevention and Treatment of *Candida* Albicans Biofilms

**DOI:** 10.3390/pharmaceutics12100918

**Published:** 2020-09-25

**Authors:** Irene Heredero-Bermejo, Natalia Gómez-Casanova, Sara Quintana, Juan Soliveri, Francisco Javier de la Mata, Jorge Pérez-Serrano, Javier Sánchez-Nieves, José Luis Copa-Patiño

**Affiliations:** 1 Department of Biomedicine and Biotechnology, Faculty of Pharmacy, University of Alcalá, 28871 Alcalá de Henares, Spain; natalia.gomezc@uah.es (N.G.-C.); juan.soliveri@uah.es (J.S.); jorge.perez@uah.es (J.P.-S.); josel.copa@uah.es (J.L.C.-P.); 2 Department of Organic and Inorganic Chemistry, Research Institute in Chemistry “Andrés M. del Río” (IQAR), University of Alcalá, 28871 Alcalá de Henares, Spain; sara.quintana@edu.uah.es (S.Q.); javier.delamata@uah.es (F.J.d.l.M.); javier.sancheznieves@uah.es (J.S.-N.); 3 Institute “Ramón y Cajal” for Health Research (IRYCIS), 28034 Madrid, Spain; 4 Networking Research Center on Bioengineering, Biomaterials and Nanomedicine (CIBER-BBN), 28029 Madrid, Spain

**Keywords:** *Candida*, antifungal drugs, *Candida albicans*, biofilm, biocides, carbosilane dendrimers, cytotoxicity, scanning electron microscopy

## Abstract

*Candida* spp. are one of the most common fungal pathogens. Biofilms formed by *Candida* *albicans* offer resistance mechanisms against most antifungal agents. Therefore, development of new molecules effective against these microorganisms, alone or in combination with antifungal drugs, is extremely necessary. In the present work, we carried out a screening process of different cationic carbosilane dendritic molecules against *C. albicans*. In vitro activity against biofilm formation and biofilms was tested in both Colección Española de Cultivos Tipo (CECT) 1002 and clinical *C. albicans* strains. Cytotoxicity was studied in human cell lines, and biofilm alterations were observed by scanning electron microscopy (SEM). Antifungal activity of the carbosilane dendritic molecules was assessed by monitoring cell viability using both established and novel cell viability assays. One out of 14 dendritic molecules tested, named BDSQ024, showed the highest activity with a minimum biofilm inhibitory concentration (MBIC) for biofilm formation and a minimum biofilm damaging concentration (MBDC) for existing biofilm of 16–32 and 16 mg/L, respectively. Synergy with amphotericin (AmB) and caspofungin (CSF) at non-cytotoxic concentrations was found. Therefore, dendritic compounds are exciting new antifungals effective at preventing *Candida* biofilm formation and represent a potential novel therapeutic agent for treatment of *C. albicans* infection in combination with existing clinical antifungals.

## 1. Introduction

*Candida* is a fungal opportunistic pathogen responsible for serious infections in humans, including candidiasis, especially in immunocompromised individuals [1,2]. Among all *Candida* species, *C. albicans* is one of the most common fungal pathogen worldwide [3]. These fungi can grow on biotic and abiotic surfaces and are associated with the formation of biofilms [4,5].

Biofilm formation has clinical implications and continues to be a major problem, contributing to increased mortality rates associated to these fungal infections. Medical devices, such as catheters or prostheses [6,7], are common niches where biofilms manage to adhere and develop, and once formed, these biofilms provide resistance to high concentrations of antifungal compounds. The composition of biofilms varies depending on the species of microorganisms, the quality of the environment, and many other variants [8,9]. Among *Candida* species*, C. albicans* is the most commonly associated with biofilm formation, being particularly important in hospitals [6,10]. The extracellular matrix is composed of self-produced polysaccharides [6,7,11], which offer protection to cells that comprise the biofilm against the host’s immune system. This matrix also blocks drugs from penetrating the biofilm and prevents antifungal compounds from reaching their target. This often results in the development of drug resistance that causes severe health problems, including bloodstream infections, in hospitalized patients [8,12]. Therefore, biofilms are an important reservoir of infections and represent a key target for the development of novel antifungal therapeutics.

Infections caused by *Candida* spp. are currently being treated with a wide variety of antifungal agents [13]. These include azoles, such as fluconazole (FLU), which inhibit ergosterol biosynthesis or amphotericin B (AmB) that impair fungal cell membrane integrity, as well as echinocandins, such as caspofungin (CSF), which act on the synthesis of 1,3-beta glucans [13,14]. However, *Candida* biofilms are resistant to many of these standard antifungal agents, and few/no molecules have yet been identified that effectively target biofilms [15]. As a result, there is a significant need for the development of new biocides to treat *Candida* biofilm-related infections.

Dendrimers have emerged as an exciting new class of microbicidal agents with low toxicity to human cells [16,17]. These macromolecules are highly branched globular structures with a central nucleus and a polyfunctional periphery [16,18]. This multivalency and the diversity of functional groups that can be introduced in it are very attractive for biomedical applications. Previous studies have reported the antibacterial [16,18,19,20,21,22] and antiamoebic [21,23] activity of these cationic dendrimers and have found that their main target is the plasma membrane of microorganisms. One type of cationic dendrimer with attractive microbicide properties is carbosilane dendrimers (CBS), which contains a framework made of weakly polar C-C and C-Si bonds [24]. CBS have been investigated for their applications as antibacterial, antifungal and antiviral agents; nanodrugs; drug delivery systems; genetic material carriers; artificial proteins; synthetic vaccinations; among others [25]. In contrast with other types of commercial dendrimers, such as poly(amido amine) (PAMAM) or poly(propylene imine) (PPI), carbosilane dendritic systems present a hydrophobic backbone that can facilitate the interaction with biological membranes. In this sense, we have observed that in general, low generation carbosilane dendrimers are suitable to get the desired biological effect. This is crucial because it has been reported that the toxicity of dendritic systems increases with the dendritic generation. One dendrimer of this type showed synergistic effect against *Acanthamoeba polyphaga* when combined with chlorhexidine digluconate [26]. Dendrimers have also been used as antifungals [27], as delivery systems for commercial antifungals [28], and are associated with commercial biocides to enhance their solubility [29,30] and/or activity [30,31].

The aim of the present study was to determine the antifungal properties of synthesized library of CBS dendritic systems to prevent biofilm formation, and to examine their activity against established *C. albicans* biofilms. Additionally, we studied possible synergies with commercial antifungal drugs, checked their cytotoxicity against human cells, and visualized the effect of dendrimers on biofilm cells using scanning electron microscopy (SEM). Our results suggest that a combination therapy using these dendritic compounds with currently available clinical antifungals may be an effective alternative treatment for candidiasis. Additionally, co-administration of dendrimers alleviates limitations of current first-line therapeutics by targeting the biofilm in a synergistic manner that reduces the potential for development of drug resistance.

## 2. Materials and Methods

### 2.1. Candida, Growth Conditions and Stimulation for Biofilm Formation

*Candida albicans* strain of Colección Española de Cultivos Tipo (CECT) 1002 and a clinical isolate of *C. albicans* from Hospital Universitario Príncipe de Asturias (Madrid, Spain) were used in this study. *Candida* isolated were stored at −80 °C with 20% glycerol (Sigma-Aldrich, Saint Louis, MO, USA) until use. The strains were grown on Sabouraud chloramphenicol agar (Scharlab, Barcelona, Spain) overnight. Then, various colonies were transferred into 45 mL of Yeast Extract (1%)–Peptone (2%)–Dextrose (2%) (YPD, Scharlab, Barcelona, Spain) to stimulate biofilm formation, and incubated with slight agitation (150 rpm) at 37 °C for 24 h.

### 2.2. Biofilm Formation Assay

The inoculum was prepared as previously described [32,33]. *C. albicans* cells were harvested and resuspended in RPMI 1640 medium (Sigma-Aldrich, Saint Louis, MO, USA) buffered with MOPS (morpholinepropanesulfonic acid, Sigma-Aldrich) and supplemented with 2% glucose (Scharlab, Barcelona, Spain) to a cellular density equivalent to 0.25, 0.5 or 1 McFarland standard (Grant Instruments, Royston, UK) (raw data in the Supplementary Materials. Tables S1 and S2. Figure S1). Finally, after performing these experiments, a 0.5 McFarland (1.5 × 10^6^ UFC/mL) was [33]). To allow biofilm formation, 100 μL of the suspension were inoculated into 96-well microtiter plates (NUNC^TM^) and incubated at 37 °C for 48 h until biofilm was formed. 

### 2.3. Biofilm Quantification 

To confirm biofilm formation and test its homogeneity, biofilm development was quantified using crystal violet method [34]. To run this method, the supernatant from each well was discarded, and the biofilm was carefully washed with 200 µL sterile 1X phosphate buffered saline (1X PBS, Sigma-Aldrich, Saint Louis, MO, USA). Wells were dried for 30 min at 37 °C. Then, 50 µL of crystal violet (1% *w/v*, Sigma-Aldrich, Saint Louis, MO, USA) were added to each well and incubated for 15 min. Wells were washed twice with sterile 1X PBS to remove unabsorbed crystal violet solution. Then, wells were destained with 200 µL of acetic acid (33% *v/v,* Scharlab, Barcelona, Spain) for 15 min and each well was shaken with the pipette. After that, 150 µL of this solution were transferred to a new 96-well plate and absorbance was read at 630 nm using a microtiter plate reader (Epoch^TM^, BioTek Instruments, Winooski, VT, USA). Control wells were included in all experiments to eliminate any background signal (free-inoculum control).

### 2.4. Cationic CBS Dendritic Molecules

**Figure 1:**
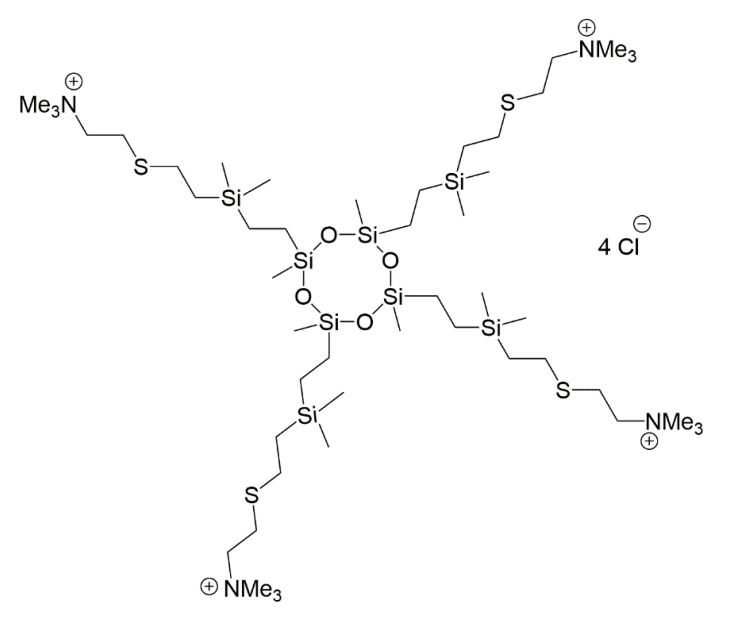
Drawing of BDSQ024 dendrimer structure.

**Scheme 1:**
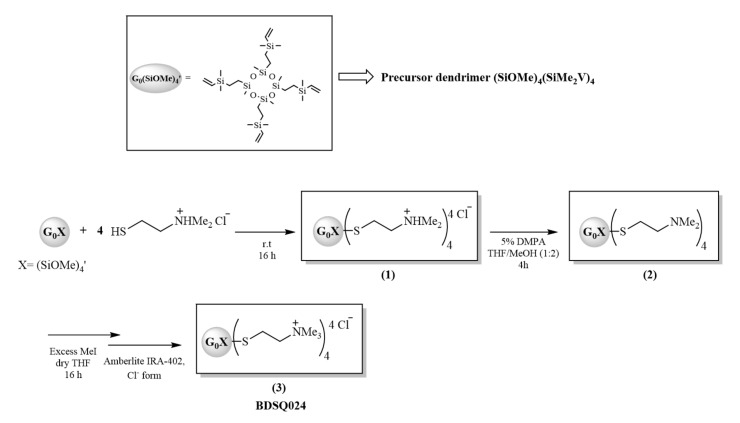
Synthetic procedure of BDSQ024.

The dendritic molecules used in the present study are shown in Table 1. Compounds of various topologies, number of positive charges, nature (dendritic molecules or dendronized nanoparticles), composition, and flexibility, were chosen to allow for testing of multiple properties that may influence the activity of these compounds. We have previously reported on the development of a subset of these CBS dendritic systems (Table 1) and one of them, BDSQ024 (Figure 1), was prepared for this study, trying to see the influence of a more open core that changes the flexibility and placed the terminal groups at a greater distance than in derivatives previously synthesized by our group. The process for preparation of BDSQ024 is illustrated in Scheme 1, with additional information provided in the Supplementary Materials (Supplementary S1) [35]. 

The ability of these molecules to prevent biofilm formation and eliminate *C. albicans* biofilm was studied. The compounds were prepared at stock concentrations of 1024 mg/L and antifungal susceptibility testing was performed using the NCCLS M27A broth microdilution reference method with reading of endpoints at 48 h [33,41]. Biocides were tested in microtiter plates using a series of two-fold dilutions with concentrations ranging from 2 to 512 mg/L. Assays were run in technical triplicate and repeated at least twice.

The ability for each compound to disrupt biofilms was examined by comparision with compound-free wells (0% disruption), and cells treated with AmB at the lethal concentration of 64 mg/L (100% disruption). The negative controls were tested on *Candida*-free wells adding medium and dendritic molecules.

### 2.5. Resazurin Colorimetric Viability Assay

Resazurin colorimetric assays were used to assess *Candida* viability after treatment and were performed as previously described, with minor modifications to set up resazurin concentration and incubation time to ensure reproducible results in our conditions [42,43]. The experiments conditions were studied to get the optimal resazurin concentration and the optimal incubation time, as these are crucial to obtain reliable data. The resazurin concentrations ranged from 0.01 to 0.5% (*w/v*), and the incubation times tested were 2, 3, 4, 5, and 6 h. All experiments were run in technical triplicate for at least two independent biological replicates (raw data of resazurin experiments in the Supplementary Materials. Tables S3–S6).

Resazurin (Sigma-Aldrich) was prepared as a 0.01% (*w/v*) solution in sterile distilled water, filtered through a 0.22-μm-pore-size filter, and stored at 4 °C. After treatment and incubation, 20 μl of the resazurin solution was added to each well containing 100 μL PBS or culture medium. Plates were incubated in the dark for 3 h at 37 °C. Live yeast cells reduced resazurin and produced a pinkish color, indicating viability. Absorbance was measured in a microplate reader (Epoch^TM^, BioTek) at 570 nm.

### 2.6. Drop Plate Method

To confirm data obtained by the colorimetric viability assay, we transferred 10 µl of each well onto chloramphenicol-Sabouraud agar plates (agar drop plate method) [44]. Additionally, 10 µl of each well were transferred to plate count agar (PCA) to assess the presence or absence of bacterial contaminants. The plates were incubated at 37 °C overnight. The minimum fungicidal concentration (MFC) is defined as the lowest concentration of compound that yielded no *Candida* growth on agar plates, and the minimum biofilm eliminating concentration (MBEC) is defined as the lowest concentration resulting in a 100% cells death of the biofilm (no growth observed on agar plates) [45,46].

### 2.7. Pre-Biofilm Treatment

A *Candida* cells suspension was adjusted to a density equivalent to 0.5 McFarland standard in RPMI 1640 medium (Sigma-Aldrich) with MOPS and 2% glucose. To run the experiments, 96-well microtiter plates containing 100 µL of biocide in a two-fold dilution series prepared in RPMI 1640 medium ranging from 4 to 1024 mg/L were innoculated with 100 µL of the *Candida* cell suspension. A biocide-free control (positive control), a treatment control (64 mg/L AmB) and un-inoculated control (negative control) were included in all experiments. Plates were sealed with Parafilm (Bemis, Neenah, WI, USA) and incubated for 48 h at 37 °C.

The minimum biofilm inhibitory concentration (MBIC) was calculated for each compound conducting the resazurin colorimetric assay. This concentration was defined as the lowest concentration that completely inhibited *Candida* growth and biofilm formation after 48 h treatment. The MFC value was obtained for the pre-biofilm experiments, as it is described in Section 2.5.

### 2.8. Biofilm Treatment

After biofilm formation (48 h incubation), growth medium was carefully aspirated and biofilms were gently washed, adding sterile 1X PBS in a drop-wise manner to remove non-adherent cells. Then, serial concentrations of dendritic compounds prepared in RPMI 1640 medium (Sigma-Aldrich) with MOPS and 2% glucose were added to a final volume of 100 µL (concentrations ranging from 2 to 512 mg/L). A biocide-free control (positive control), a treatment control (64 mg/L AmB) and un-inoculated control (negative control) were included in all experiments. Plates were sealed with Parafilm^®^ and incubated for 48 h at 37 °C. The most effective dendrimers were studied further. In this case, the biofilm was treated again with the same serial concentration of dendrimer and incubated for another 24 h (72 h total period).

After treatments, the minimum biofilm damaging concentration (MBDC) was determined using the resazurin colorimetric method, and the minimum biofilm eliminating concentration (MBEC) by the agar plating method. The MBDC was defined as the lowest concentration that caused damage and affected or inhibited *Candida* metabolic activity.

### 2.9. Synergistic Activity of New Dendrimers and Commercial Antifungal Drugs against *Candida albicans*

Synergistic activity was studied against *C. albicans* cells, testing for both inhibition of biofilm formation, and for activity against established biofilms using the checkerboard titration technique [47]. Amphotericin (AmB) and caspofungin (CSF) were used as reference antifungal drugs. For biofilm pre-treatments, 0.007 to 0.5 mg/L of antifungals were used, while existing biofilms were treated with 0.03 to 2 mg/L (AmB) and 0.03 to 16 mg/L (CSF). A concentration of 32 mg/L of BDSQ024 was used for biofilm pre-treatment, while 64 mg/L was used to treat existing biofilm. To obtain the values of MBIC and MBDC, the same methodology was performed as described above. Plates were incubated for 48 h at 37 °C.

Fractional inhibitory concentration index (FICI) was calculated for the interpretation of combination effects. FICI = (MBIC (or MBDC) drug A combination with B/MBIC (or MBDC) drug A alone) + (MBIC (or MBDC) drug B combination with A/MBIC (or MBDC) drug B alone). A FICI ≤ 0.5 meant synergy; FICI > 0.5 to ≤ 2.0, indifference; and FICI > 2.0 meant antagonism [47].

### 2.10. Cytotoxicity of CBS Dendrimers and Commercial Antifungal Drugs in Hela and Human Foreskin Fibroblasts Cells

The cytotoxicity was evaluated on using HeLa (ATCC^®^ CCL-2™) and Human Foreskin Fibroblasts (HFF) (ATCC-SCRC-1041). Experiments were performed in 24-well plates (NUNC^TM^) in Dulbecco’s Modified Eagle Medium (DMEM) supplemented with 10% foetal bovine serum (Sigma-Aldrich Ltd.) and 1% antibiotic mix: 10.000 U penicillin, 10 mg streptomycin and 25 µg AmB per mL (Sigma-Aldrich Ltd.). Cells were seeded at a density of 1 × 10^4^ cells/well in 500 µL of fresh medium. Then, plates were incubated for 5 days at 37 °C in a 5% CO_2_ atmosphere to form a confluent cell monolayer. After incubation, the medium was replaced by 200 µL of fresh medium plus 200 µL of drugs/dendrimers. Control wells received 200 µL of distilled water and 200 µL of fresh medium. After 48 h of incubation, the culture medium was discarded, wells were washed three times with 1X PBS to eliminate any residual drug, and 500 µL of fresh medium were added to each well. Cytotoxicity was evaluated using the Microculture Tetrazolium Assay (MTT, Sigma-Aldrich Ltd.). Each well received 50 µL of MTT (5 mg/mL) and plates were incubated for w4 h at 37 °C. Subsequently, medium was discarded and 500 µL of dimethyl sulfoxide (DMSO) were added to dissolve formazan crystals. Absorbancas e of the samples recorded in a microplate absorbance reader at 570–630 nm (BioTek Instruments Inc. Model: ELX 800). Assays were performed in triplicate for each concentration and repeated at least twice. Cytotoxicity values lower than 10% were considered non-cytotoxic. Values between 10% and 25% were considered low cytotoxicity and values from 25% to 40% were considered moderate cytotoxicity levels [26].

### 2.11. Scanning Electron Microscopy

Scanning electron microscopy (SEM) was performed to evaluate biofilm morphological differences between biofilms treated with vehicle control or dendrimer BDSQ024. *C. albicans* was grown on a glass coverslip and fixed in Milloning’s solution containing 2% glutaraldehyde for 24 h. Then, cells were washed in Milloning’s solution with 0.5% glucose and dehydrated first through an ethanol series and finally with anhydrous acetone. Samples were critical-point dried using a Polaron CPD7501 critical-point drying system, and sputter-coated with 200 Ǻ gold-palladium using a Polaron E5400. Scanning electron microscopy was performed at 5–15 kV in a Zeiss DSM 950 SEM.

### 2.12. Statistical Analysis

Each experiment was performed in triplicate and repeated at least twice. One-way analyses of variance (ANOVA) and Tukey’s test were performed. *p* < 0.05 values were considered statistically significant. All analyses were done using the GraphPad Prism 8.4 program for Windows (GraphPad Software, 2020, San Diego, CA, USA).

## 3. Results

### 3.1. Optimal Biofilm Formation

Our initial experiments using the crystal violet methodology showed that biofilm formation occurs at similar levels when inoculums between 0.5 and 1.0 McFarland standards are used. In contrast, we observed that inoculation with 0.25 McFarland standards resulted in poor biofilm formation. Therefore, the 0.5 McFarland standard was selected as the inoculum to form biofilms in further experiments.

### 3.2. Resazurin Viability Assay

The resazurin colorimetric method was selected to quantify biofilm viability. After performing experiments using different resazurin concentrations and incubations times, it was concluded that optimal conditions were obtained using a resazurin concentration of 0.01% and incubating for 3 h. These conditions were chosen to perform our experiments.

### 3.3. Dendrimer Activity Preventing Biofilm Formation

Among the 14 compounds tested, the two compounds that most effectively prevented biofilm formation were BDSQ024, a spherical dendrimer with four trimethyl ammonium groups on the surface and a siloxane core that gives great flexibility to this molecule; and YF017, a carbosilane dendron also with four terminal trimethyl ammonium groups. For *C. albicans* CECT 1002, the MBIC value reached after 48 h treatment was between 16–32 mg/L (BDSQ024) and 32 mg/L (YF017), using resazurin viability assay (Table 2). In agreement with these results, assessment of the MFC value using growth on agar plates was 32 mg/L with each compound. The compound BDSQ024 was also tested against a clinical *C. albicans* strain and observed lower MBIC and MFC values (MBIC and MFC: 8 mg/L) than *C. albicans* CECT 1002 (MBIC: 16–32 mg/L and MFC: 32 mg/L). These results show that BDSQ024 has activity against *C. albicans* and prevents biofilm formation at concentrations between 8–32 mg/L.

### 3.4. Dendrimer Activity Against Biofilms

Among the compounds tested against pre-existing biofilms, the most effective were BDSQ024 and YF017 (Table 2). Both compounds were used to study the MBDC and MBEC against biofilms of *C. albicans* CECT 1002. The antifungal activity of BDSQ024 (16 mg/L) was higher than the activity of YF017 (128 mg/L). Therefore, only BDSQ024 was selected for further experiments.

For both *C. albicans* strains used in this study, the MBDC value reached using compound BDSQ024 after 48 h treatment was 16 mg/L. However, growth was observed at this concentration and even at higher concentrations when transferring biofilm suspensions onto agar plates. These experiments showed that the concentration capable to eliminate the biofilm at 48 h was over 512 mg/L for both *C. albicans* strains (MBED > 512 mg/L). Therefore, results obtained only by colorimetric assays, such as resazurin, should not be independent from agar plating to assess biofilm cell viability when determining the ability of compounds to eliminate biofilm.

We next tested whether an additional 24 h of treatment would provide better inhibition of growth on agar plates but found that extending treatment time to 72 h provided no additional benefit beyond 48 h treatment. The MBDC value reached after 72 h treatment using resazurin viability assay was found to be identical to the value obtained when only treating for 48 h (16 mg/L). In addition, we did not get the MBEC values as we observed growth at this concentration and even higher concentrations when plating samples on agar plates. Therefore, these results show that although these compounds are having some effect on existing biofilms, they are not able to completely kill cells in the biofilm.

### 3.5. ANF and CSF: Antifungal Susceptibility on Pre-Biofilms and Biofilms

The MBIC values of individual antifungal drugs against pre-biofilms were 0.25 mg/L for AmB (MFC: 0.25 mg/L) and 0.25–0.5 mg/L for CSF (MFC: 1 mg/L). For treatment of existing biofilms, AmB showed a MBDC value of 0.5 mg/L (MBEC: 4 mg/L). However, we were not able to determine the MBDC value for CSF using resazurin assay because, performing this colorimetric assay, growth signal was observed for all concentrations tested. On the other hand, when *C. albicans* treated suspensions were grown on agar plates, the MBEC value was determined for CSF (8 mg/L).

### 3.6. Synergistic Activity of BDSQ024 and Commercial Antifungals Combinations against *Candida albicans* CECT 1002

BDSQ024 was used to study synergy in combination with CSF or AmB. Combining 8 mg/L of BDSQ024 (reduction from 16–32 mg/L. *p* < 0.01) managed to reduce AmB MBIC value from 0.25 to 0.06 mg/L (*p* = 0.0246) (FICI: 0.49—low synergy) (Table 3). Even greater synergy was observed between BDSQ024 and CSF: 8 mg/L. In this case, two combinations showed synergy: 8 mg/L BDSQ024 (reduction from 16–32 mg/L. *p* < 0.01) in combination managed to reduce the CSF MBIC value from 0.25–0.5 to 0.007 mg/L (*p* < 0.001) (FICI value: 0.26), and 4 mg/L BDSQ024 (reduction from 16–32 mg/L. *p* < 0.0001) in combination managed to reduce the CSF MBIC value from 0.25–0.5 to 0.03 mg/L (*p* = 0.01) (FICI value: 0.18) (Table 3). Therefore, these combinations allowed a significant reduction in BDSQ024 CBS dendrimer and antifungals concentrations compared to individual data treatments.

On the other hand, no synergistic combination was found that eliminated biofilm in its entirety, as *C. albicans* biofilm suspensions always grew on agar plates (MBEC) at combinations tested. Nonetheless, a notable reduction in growth was observed on agar plates at the highest concentrations for both compounds’ combinations comparing to data obtained on individual treatments (BDSQ024/AmB and BDSQ024/CSF combinations).

### 3.7. Cytotoxicity of AmB and CSF

**Figure 2:**
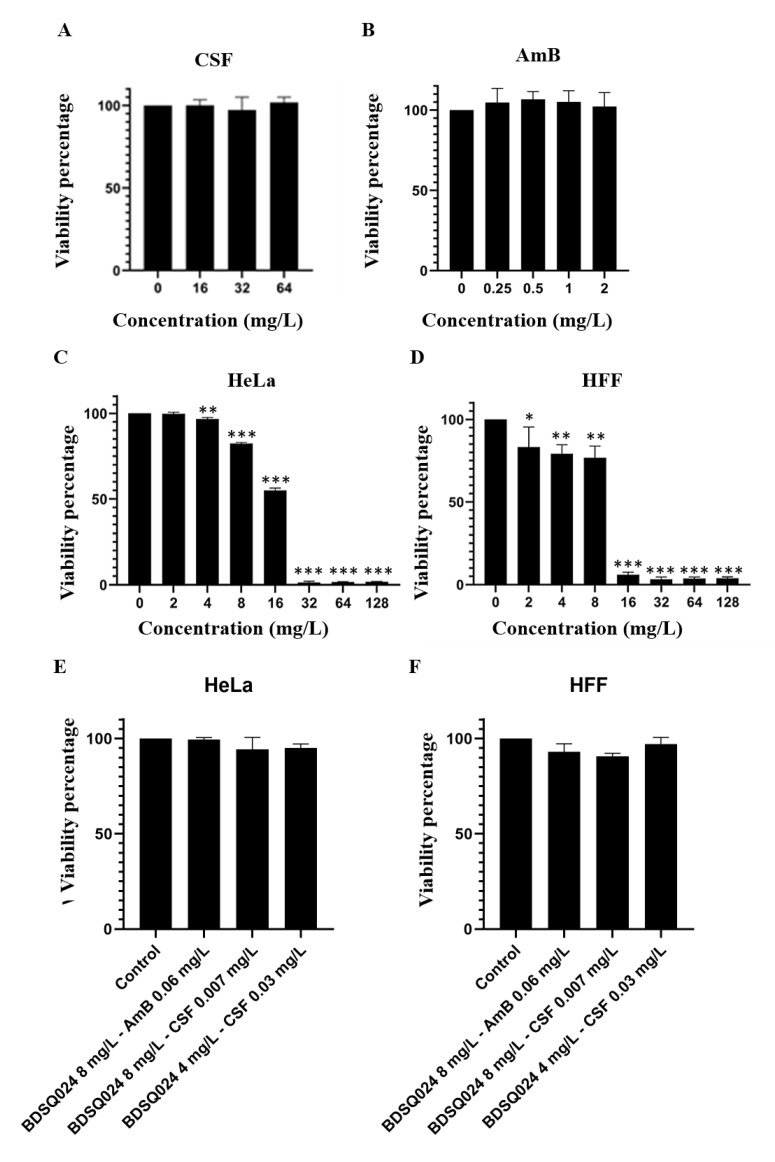
Cytotoxicity of antifungals (CSF and AmB), BDSQ024 and combinations on human cell lines. (**A**) CSF; (**B**) AmB; (**C**) BDSQ024 on HeLa cells; (**D**) BDSQ024 on HFF cells; (**E**) combinations on HeLa cells; (**F**) combinations on HFF cells. *p*-values (comparison to control): * *p* < 0.05, ** *p* < 0.001, *** *p* < 0.0001.

Reference drugs used in our experiments were not cytotoxic against Hela and HFF cells at any of the concentrations studied (Figure 2A,B). Cell viability was always 100% for all concentrations tested.

### 3.8. Cytotoxicity of BDSQ024 on Hela and HFF

BDSQ024 showed high cytotoxicity at MBIC concentrations (Figure 2C,D). This dendrimer produced almost 100% cell viability reduction at 32 mg/L on Hela cells and at 16 mg/L on HFF cells. MBIC for BDSQ024 is between 16–32 mg/L, which means that these concentrations are toxic for both cell lines.

The combinations BDSQ024/AmB and BDSQ024/CSF that show synergistic effect were also tested on HeLa and HFF cell lines to check their cytotoxicity. The results showed that the dendrimer in combination with AmB or CSF has no cytotoxicity against human cell lines (Figure 2E,F). The MBICs for this dendrimer in combination with AmB and CSF were 8 and 4 mg/L, respectively. Therefore, we observed that cytotoxicity was lower when testing in combination and cell viability was never lower than 80%.

### 3.9. Scanning Electron Microscopy: Observations on the Biofilm Morphology

**Figure 3:**
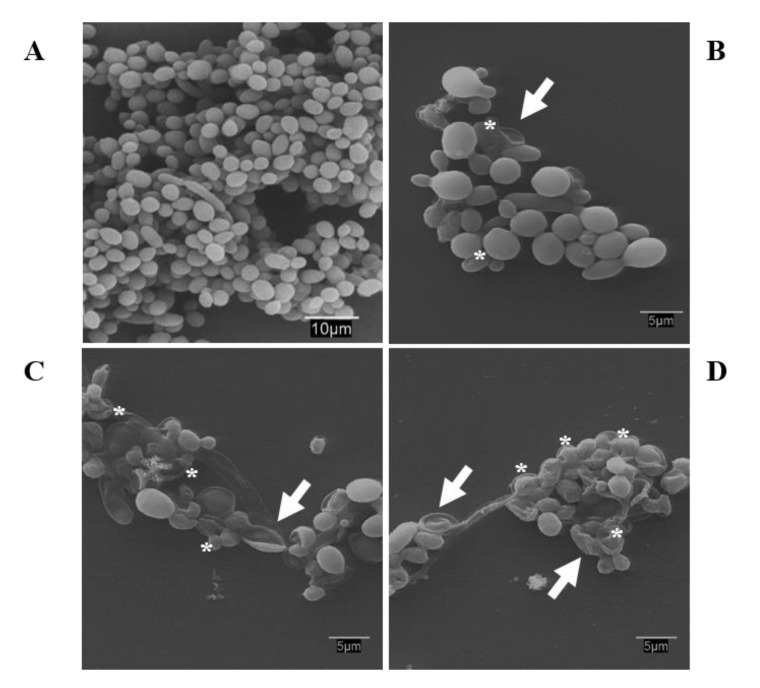
Alterations produced on *C. albicans* CECT 1002 biofilm with BDSQ024 dendrimer observed by SEM. 48 h treatment. (**A**) Control untreated; (**B**) 16 mg/L; (**C**) 32 mg/L; (**D**) 64 mg/L. Arrows: collapsed cells. Asterisks: perforations on cell membrane.

A homogeneous biofilm was verified in the control samples, with rounded-shape cells and different cell layers that formed the biofilm (Figure 3A). However, biofilm density was reduced by BDSQ024 dendrimer action, with cells forming a monolayer with dispersion between biofilm cells (Figure 3B,C). Also, treated biofilms showed alterations to *C. albicans* CECT 1002 cell morphology and perforations on the outer membrane (Figure 3B–D, asterisks), especially at higher concentrations. At the concentration of 32–64 mg/L, cell shrinkage was observed (Figure 3C,D, arrows).

## 4. Discussion

*C. albicans* is an opportunistic pathogen of global clinical significance [48,49,50]. Preventing biofilm formation and eradicating existing biofilms is important to reduce mortality rates of infections caused by these microorganisms. Unlike *Candida* planktonic cells, in the biofilm state, cells have a greater ability to evade the effects of antifungals and are resistant to treatment [51]. Additionally, the range of antifungal agents used clinically is limited and the development of drug resistance is a growing concern. Therefore, it is crucial to develop new agents effective against *Candida*. In this study, we analyzed the fungicidal and fungistatic ability of different types of CBS dendritic compounds, including several nanoparticles and dendrimers with copper complexes, against *C albicans* biofilm formation and biofilms. One of these compounds, BDSQ024, a generation 0 dendrimer containing a tetrasiloxane ([SiO]_4_) core from which four branches are growing and contains four -NMe_3_^+^ terminal groups on the periphery, showed the highest antifungal activity preventing biofilm formation, as well as against biofilm. The carbosilane skeleton of this dendritic system provides a high stability, making these compounds have a high chemical inertness that prevents the appearance of secondary reactions. The flexibility that induces this new core, together with the hydrophilic/hydrophobic balance present in its structure, could lead to the high activity observed for this compound. Regarding the compound size, it has been reported that a higher dendrimer generation did not always correlate with a higher activity. Instead, a suitable hydrophilic/hydrophobic balance seems to be more important in order to establish the activity of these carbosilane dendritic derivatives [52].

Among methods used to assess biofilm formation, crystal violet method is among the most common used [53,54,55]. This method allows for the quantification of total biofilm biomass; however, it does not provide a measure of biofilm viability, as even dead cells and extracellular matrix can incorporate the reagent. Therefore, a method to assess fungal viability is essential. For that purpose, we selected the resazurin colorimetric assay, which uses a phenoxazine dye that is non-toxic and redox-sensitive. Unlike other colorimetric indicators, resazurin does not require the use of DMSO to dissolve salts to reveal viability results. This metabolic method is similar to tetrazolium assays, such as MTT (3-(4,5-dimethylthiazol-2-yl)-2,5-diphenyltetrazolium bromide) and XTT (2,3-bis-(2-methoxy-4-nitro-5-sulfophenyl)-2H-tetrazolium-5-carboxanilide), which have been previously used to measure cell viability in [15,33,56]. Data obtained performing the resazurin colorimetric assay was reliable for the determination of the MBIC and MBDC results. However, to assess the MFC and MBEC values, the agar plating method was required, as discussed below. The reduction of metabolic rates and cellular respiration in biofilms is well known, so there may be metabolically latent cells [47,57,58]. This fact may explain the absence in the reduction of the dye in some assays. Nevertheless, some cells remained viable in the biofilm and proliferate when transferring suspension onto agar plates. Therefore, unlike Ravi et al. [45], in our study, use of the resazurin colorimetric assay alone did not allow us to obtain reliable MBEC values in existing biofilms. These data suggested that some biofilm cells remain dormant due to cell stress, preventing the reduction of resazurin. For example, antimicrobial resistance has been associated with a metabolic reduction in bacteria biofilms due to decreased nutrients and ATP in these conditions (persister cells) [57,58]. In the case of *Candida*, these limited subpopulations of persister cells are responsible for the antifungal resistance [59,60,61] and remain metabolically latent [7]. Collectively, our data suggest that the use of these metabolic assays to quantify biofilm viability could underestimate the real viability percentage and could report concentration values that did not eliminate 100% of biofilm cells. The agar drop plate method is widely used in studies with planktonic cells. However, in studies performed to quantify biofilm viability using some cell respiration detection agents, the agar drop plate method is not used. In this study, we have found that the agar drop plate method is not only complementary to determinate the MFC, but also essential to determine reliable MBEC values in the stablished incubation time.

The data obtained in this study showed that BDSQ024 had a fungistatic effect against biofilm and severely damaged *C. albicans* cells as clearly observed using SEM. Figure 3A showed untreated *C. albicans* CECT 1002 dense biofilms structures containing spherically-shaped cells with typical budding and smooth walls [62,63,64]. On the other hand, treated cells showed alterations on cellular morphology, alterations on the uniformity of biofilm layers, a reduction in the number of cells, and a significant increase in the number of enlarged blastosphores; especially from concentrations of 32 mg/L. Further, collapsed *C. albicans* cells were also observed (Figure 3B,D). Our observations are in agreement with previous reports [62,63] and suggest that the cationic dendrimer BDSQ024 may be interacting with the negatively-charged cell membrane, resulting in ion leakage, permeability and compromising membrane integrity. This alteration may ultimately cause membrane depolarization, which ends in collapsed and dead cells. Additionally, dendrimers may penetrate into the cell and may affect organelles and cell processes vital for cell survival.

The most interesting results showed that treatments using a combination of BDSQ024 dendrimer and commercial antifungal drugs had a synergistic reduction in the effective concentration values obtained in the pre-biofilm treatment assay, where the concentrations of both antifungals and BDSQ024 were significantly reduced to avoid biofilm formation. The concentration of AmB was decreased to 0.06 mg/L and CSF to 0.007 mg/L. In the case of BDSQ024 dendrimer, it was reduced to a non-cytotoxic concentration of 4 mg/L. Reducing the doses of treatment with antifungal drugs could reduce the likelihood for emergence of drug resistant strains, and could result in less aggressive, better tolerated treatment regimens for the patient. Indeed, co-treatment with BDSQ024 and AmB or CSF was effective at concentrations that were non-cytotoxic to HeLa and HFF cells. Therefore, the combinations of antifungal drugs with BDSQ024 showed a synergistic activity and a decrease on cytotoxicity comparing to individual drug treatment, as reported by other researchers [65]. These combinations would be an interesting therapeutic approach to prevent biofilm formation. In fact, the effect of these combinations on preventing biofilm formation may be remarkable for medical device applications, because biofilm formation can lead to severe complications in hospitalized patients [12], and for topical solutions to eliminate *Candida* cells.

Towards this goal, we will focus on the synthesis of new molecules based on the structure of the effective compound BDSQ024, the design of strategies to better understand the mechanism of action of these compounds, and the combined therapy with other antifungal drugs to completely eliminate biofilms.

## 5. Conclusions

In this study, we found an effective cationic carbosilane dendritic molecule, BDSQ024, capable of eliminating *C. albicans* at low concentrations and damaging existing biofilms. Further, we confirmed that when performing biocide assays against existing biofilms, it is necessary to plate well suspension on agar plates to determine MBEC values. These assays must be done in parallel with the colorimetric viability assays due to the presence of metabolically inactive cells that retain the capacity to regenerate the biofilm. Finally, combinations of commercial antifungal drugs with BDSQ024 resulted in an important decrease in effective concentrations and cytotoxicity, and an increase in the susceptibility of *Candida* cells to antifungals. Therefore, these combinations may be an interesting therapeutic approach to control biofilm formation by generating antiseptic topical solutions containing both substances, with low concentrations of commercial antifungals.

## Figures and Tables

**Table 1:** Characteristics of dendritic compounds tested against *C. albicans*.

Lab Name	Compound	Core	Functional Groups	R
BDSQ024	C_48_H_120_ Cl_4_N_4_O_4_S_4_Si_8_	(SiOMe)_4_	4, NMe_3_^+^	-
AG3	C_64_H_160_Cl_8_N_8_O_4_S_8_Si_8_	(SiOMe)_4_	8 NMe_2_H^+^	[36]
BDAB24	HSG1(SNMe_3_^+^)2:HSHPEG, ratio 1:1	AgNP	-NMe_3_^+^, -PEG	[37]
BDAB27	HSG2(SNMe_3_^+^)4:HSHPEG, ratio 1:1	AgNP	-NMe_3_^+^, -PEG	[37]
BDAB28	HSG3(SNMe_3_^+^)4:HSHPEG, ratio 3:1	AgNP	-NMe_3_^+^, -PEG	[37]
BDAT2	C_56_H_100_Cu_4_N_16_O_28_Si_5_	Si	4 pyridine-imine-Cu	[38]
BDLS001	C51H114Cl6N18O3S6Si3	C_6_H_3_O_3_	(NHC(NH)NH_2_)_6_ (6 Guanidine)	[21]
BDEF134	C47H102I4N4O2S4Si3	Dendron	F.P.: HOC_6_H_4_O; P: 4 NMe_3_^+^	[20]
BDEF135	C91H206I8N8O2S8Si7	Dendron	F.P.: HOC_6_H_4_O; P: 8 NMe_3_^+^	[20]
ChG2	C69H145I4N5O2S4Si3	Dendron	F.P: Cholesterol; P: 4 NMe_3_^+^	[39]
ChG3	C121H265I8N9O2S8Si7	Dendron	F.P: Cholesterol; P: 8 NMe_3_^+^	[39]
EG2	C70H146I4N4O2S4Si3	Dendron	F.P: E vitamin; P: 4 NMe_3_^+^	[39]
EG3	C122H266I8N8O2S8Si7	Dendron	F.P: E vitamin; P: 8 NMe_3_^+^	[39]
YF017	C41H91Cl4N5O2S4Si3	Dendron	F.P: Maleimide; P: 4 NMe_2_H^+^	[40]

F.P.: focal point. P: peripherial groups. R: reference.

**Table 2:** Minimum biofilm inhibitory concentration (MBIC) and minimum fungicidal concentration (MFC) for all the dendritic compounds against *Candida albicans* CECT 1002.

Compounds	Biofilm Formation (mg/L) Range: 2–512 mg/L	
	MBIC	MFC
BDSQ024 *	16–32	32
AG3	128	128
BDAB24	>512	>512
BDAB27	128	512
BDAB28	256	>512
BDAT2	128	128
BDLS001	32	64
BDEF134	32–64	>512
BDEF135	128	>512
ChG2	>512	>512
ChG3	512	>512
EG2	512	>512
EG3	>512	>512
YF017*	32	32

* Only dendrimers with a MFC ≥ 32 mg/L were tested to determine their MBDC.

**Table 3:** FICI of amphotericin (AmB)/BDSQ024 dendrimer and caspofungin (CSF)/BDSQ024 dendrimer combinations against *C. albicans*. Pre-biofilm 48-h treatment.

MBICs (mg/L)				FICI	Relationship
BDSQ024 Individual	AmB Individual	BDSQ024 in Combination with AmB	AmB in Combination with BDSQ024		
16–32	0.25	8	0.06	0.49	Low synergy
16–32	0.25	4	0.125	0.62	Non-synergy
**BDSQ024 individual**	**CSF individual**	**BDSQ024 in combination with CSF**	**CSF in combination with BDSQ024**	**FICI**	**Relationship**
16–32	0.25–0.5	8	0.007	0.26	Synergy
16–32	0.25–0.5	4	0.03	0.18	Synergy

## Supplementary Materials

The following are available online at https://www.mdpi.com/1999-4923/12/10/918/s1, Suplementary material S1: Synthesis of BDSQ024 dendrimer, Table S1:Biofilm quantification: crystal violet (1% w/v). Absorbance values, Table S2: Different McFarland inoculum. Absorbance values 570 nm, Table S3: Resazurin concentrations. Absorbance values 570 nm. Table S4: Incubation times, inoculum 0.5 MF, measure time studies. Resazurin 0.01%, Table S5: Candida cells viability—resazurin assay (incubation time 3 h). In triplicate and repeated in 2 independent experiments, Table S6: *Candida* cells viability—resazurin assay (incubation time 3 h). In triplicate and repeated in 2 independent experiments. Figure S1. Crystal violet assay to assess biofilm formation in a microtiter plate.
