# Supplementary material for: In Vitro Activity of Carbosilane Cationic Dendritic Molecules on Prevention and Treatment of Candida Albicans Biofilms
Source: Pharmaceutics. 2020 Sep 25;12(10):918. doi: 10.3390/pharmaceutics12100918 (PMC7601597; doi:10.3390/pharmaceutics12100918)
Supplement: Supplementary file 1 [file pharmaceutics-12-00918-s001.pdf]

pharmaceutics

MDPI

Supplementary Materials: In Vitro Activity of Carbosilane Cationic Dendritic Molecules on Prevention and Treatment of Candida Albicans Biofilms

Irene Heredero-Bermejo†, Natalia Gómez-Casanova†, Sara Quintana, Juan Soliveri, F. Javier de la Mata, Jorge Pérez-Serrano, Javier Sánchez-Nieves and José Luis Copa-Patiño.

† These authors have contributed equally to the work.

Supplementary material S1. Synthesis of BDSQ024 dendrimer

Experimental Section

General Considerations

All reactions were carried out under inert atmosphere. If necessary, solvents were purified from appropriate drying agents. NMR spectra were recorded on a Varian Unity VXR-300 (300.13 (^{1}H), 75.47 (^{13}C) MHz) or on a Bruker AV400 (400.13 (1H), 100.60 (^{13}C) MHz). Chemical shifts (δ) are given in ppm. ^{1}H and ^{13}C resonances were measured relative to solvent peaks considering TMS = 0 ppm. UV reactions were done using a novaLIGHT TQ150-Z0 (UV-Peschl). Elemental analyses were performed on a LECO CHNS-932. Mass Spectra were obtained from an Agilent 6210 (ESI) and a Bruker Ultraflex III (MALDI-TOF). Compounds Karstedt's Pt catalyst, 2-(dimethylamino)ethanethiol hydrochloride, DMPA, NaH(CO_3),MeI were obtained from commercial sources. Compound $(SiOMe)_4(SiMe_2V)_4$ was synthesized as published [1].

Synthesis of compounds

$(SiOMe)_4(SiMe_2\text{-}NHMe_2Cl)_4$ (3): Compound **3** was prepared from the precursor dendrimer, $(SiOMe)_4(SiMe_2V)_4$. 2-(Dimethylamino)ethanethiol hydrochloride (0.44 g, 2.9 mmol) and DMPA (5%) were added to a THF/MeOH (1:2) solution of $SiOMe)_4(SiMe_2V)_4$ dendrimer (0.50 g, 0.72 mmol). The reaction mixture was deoxygenated and irradiated with ultraviolet light for 4 h. The end of reaction was checked by ^{1}H-NMR, observing that signals of vinyl groups had disappeared completely. Then, solvents were removed by rotary evaporation and compound **3** was diluted in water. DMPA is insoluble in water, and consequently, it could be removed with 0.22 µm syringe filter, whilst the excess of 2-(dimethylamino)ethanethiol hydrochloride was removed by dialysis (membrane of 100-500 Da). After removal of volatiles, compound **3** was obtained as yellow solid (0.719 g). Data for **3**: ^{1}H-NMR (D_2O): δ= -0.02 (s ancho, 36H, Si*Me*$_2$, SiO*Me*), 0.40 (m, 16H, $SiCH_2CH_2Si$), 0.81 (m, 8H, $SiCH_2CH_2S$), 2.63 (m, 8H, $SiCH_2CH_2S$), 2.84 (m, 32H, SCH_2CH_2N, N^+HMe_2), 3.29 (m, 8H, SCH_2CH_2N). ^{13}C{^{1}H} NMR (D_2O): δ= - 4.9 ($SiMe_2$), -4.0 (SiO*Me*), 6.29 ($SiCH_2CH_2Si$), 8.99 ($SiCH_2CH_2Si$), 14.9 ($SiCH_2CH_2S$), 25.5 (SCH_2CH_2N), 26.9 ($SiCH_2CH_2S$), 42.6 (N^+Me_3), 56.3 (SCH_2CH_2N). $C_{44}H_{112}Cl_4N_4O_8S_4Si_8$ (1256,12 g/mol). Calc.: C, 42.07; H, 8.99; N, 4.46; S, 10.21. Obt.: C, 40.13; H, 7.83; N, 4.11; S, 9.69. MS: 555.2766 $[M\text{-}2HCl\text{-}2Cl^-]^{2+}$.

$(SiOMe)_4(SiMe_2\text{-}NMe_2)_4$ (4): Excess of NaH(CO_3) was added to the distilled water solution of compound **3**. The reaction mixture was stirred for 1 hour at room temperature. Next, the aqueous phase was extracted twice with CH_2Cl_2 and Na_2SO_4 was used as dessicant of organic phase. Solvent was removed by rotatory evaporation yielding **4** as yellowish oil (0.244 g). Data for **4**: ^{1}H-NMR ($CDCl_3$): δ= -0.04 (s, 24H, $SiMe_2$), 0.04 (s, 12H, SiO*Me*), 0.33-0.45 (m, 16H, $SiCH_2CH_2Si$), 0.84 (m, 8H, $SiCH_2CH_2S$), 2.21 (s, 24H, NMe_2), 2.43-2.64 (m, 24H, $SiCH_2CH_2S$, SCH_2CH_2N, SCH_2CH_2N). ^{13}C{^{1}H} NMR: δ= -3.61 ($SiMe_2$), 1.08 (SiO*Me*), 6.62 ($SiCH_2CH_2Si$), 9.38 ($SiCH_2CH_2Si$), 15.88 ($SiCH_2CH_2S$), 28.07 ($SiCH_2CH_2S$), 29.94 (SCH_2CH_2N), 45.56 (NMe_2), 59.49 (SCH_2CH_2N). $C_{44}H_{108}N_4O_4S_4Si_8$ (1110,29 g/mol). Calc.: C, 47.60; H, 9.80; N, 5.05; S, 11.55; Obt.: C, 47.65; H, 9.251; N, 4.935; S, 10.985. MS: 555.2777 $[M+2H]^{2+}$.

$(SiOMe)_4(SiMe_2\text{-}NMe_3Cl)_4$ (5): Compound **4** (0.181 g, 0.160 mmol) was diluted in dry THF and mixed with excess of MeI (0.06 mL, 0.980 mmol), under an inert atmosphere. The reaction mixture was stirred for 16 hours at room temperature. Afterwards, solvent was removed under vacuum. The product was dissolved in distilled water and Amberlite IRA-402, Cl^- form was added to exchange anions. The solution was filtered, and the solvent removed under vacuum to yield compound **5** as orange solid (0.183 g). Data for **5**: ^{1}H-NMR (D_2O): δ= -0.03 (s ancho, 36H, Si*Me*$_2$, SiO*Me*), 0.41 (m, 16H, SiC*H*$_2$C*H*$_2$Si), 0.81 (m, 8H, SiC*H*$_2$CH$_2$S), 2.63 (m, 8H, SiCH$_2$C*H*$_2$S), 2.90 (m, 8H, SC*H*$_2$CH$_2$N), 3.09 (s, 36H, N$^+$*Me*$_3$), 3.47 (m, 8H, SCH$_2$C*H*$_2$N). ^{13}C{^{1}H} NMR (D_2O): δ= - 4.20 (Si*Me*$_2$), -1.66 (SiO*Me*), 6.17 (SiCH$_2$CH$_2$Si), 8.94 (SiCH$_2$CH$_2$Si), 15.04 (SiCH$_2$CH$_2$S), 24.00 (SiCH$_2$CH$_2$S), 27.57 (SCH$_2$CH$_2$N), 52.95 (N$^+$*Me*$_3$), 65.49 (SCH$_2$CH$_2$N). $C_{48}H_{120}Cl_4N_4O_4S_4Si_8$ (1312.23 g/mol). Calc.: C, 43.93; H, 9.22; N, 4.27; S, 9.77; Obt.: C, 39.40; H, 8.978; N, 4.307; S, 8.496 (traces of iodine).

Biofilm Quantification and Biofilm Formation

Table S1. Biofilm quantification: crystal violet (1% w/v). Absorbance values.

	0.25 MF	0.25 MF	0.25 MF	0.5 MF	0.5 MF	0.5 MF	1 MF	1 MF	1 MF	
**Absorbance (1)**	0.096	0.242	0.078	0.337	0.126	0.133	0.159	0.46	0.259	630nm
**Absorbance (2)**	0.056	0.069	0.056	0.163	0.173	0.161	0.221	0.177	0.251	630nm

Figure S1. Crystal violet assay to assess biofilm formation in a microtiter plate.

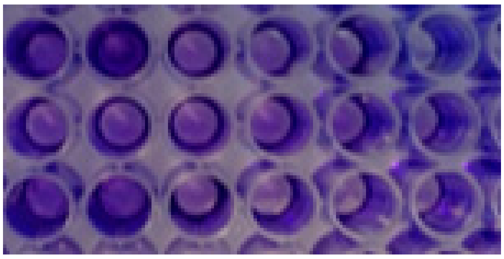

Table S2. Different McFarland inoculum. Absorbance values 570 nm.

a) Experiment 1

**1**	**1.227**	**1.121**	**1.068**	**570nm**
**0.5**	1.118	1.045	1.137	570nm
**0.25**	1.079	1.045	1.060	570nm
**Medium**	0.629	0.643	0.684	570nm

b) Experiment 2

1	**1.165**	**0.963**	**0.96**	**570nm**
**0.5**	1.249	1.251	1.076	570nm
**0.25**	1.093	0.992	0.969	570nm
**Medium**	0.678	0.649	0.654	570nm

Resazurin data

Table S3. Resazurin concentrations. Absorbance values 570 nm

	Resazurin %				
2 h incubation	**0.5**	0.944	0.999	0.914	570 nm
	**0.1**	0.781	0.867	0.989	570 nm
	**0.05**	0.883	0.998	1.039	570 nm
	**0.01**	0.872	0.803	0.805	570 nm
3 h incubation	**0.5**	1.112	1.131	1.018	570 nm
	**0.1**	1.183	1.043	1.201	570 nm
	**0.05**	0.96	1.047	1.155	570 nm
	**0.01**	0.999	0.954	1.045	570 nm

Incubation times

Table S4. Incubation times, inoculum 0.5 MF, measure time studies. Resazurin 0.01%.

a) Experiment 1

Time	Absorbance						
0h	0.854	0.840	0.697	0.718	0.816	0.708	570nm
2h	0.944	0.853	0.879	0.878	0.883	0.886	570nm
3h	1.063	0.979	1.016	1.022	1.024	1.048	570nm
4h	1.23	1.187	1.218	1.189	1.18	1.186	570nm
6h	1.391	1.368	1.352	1.39	1.387	1.378	570nm
24h	1.353	1.252	1.419	1.375	1.506	1.505	570nm
PBS 0h	0.817	0.787	0.753	0.713	0.810	0.699	570nm
PBS 2h	0.820	0.803	0.805	0.808	0.812	0.78	570nm
PBS 3h	0.812	0.806	0.800	0.802	0.815	0.786	570nm
PBS 4h	0.797	0.795	0.791	0.793	0.807	0.784	570nm
PBS 6h	0.798	0.791	0.788	0.802	0.812	0.793	570nm
PBS 24h	0.838	0.848	0.862	0.857	0.852	0.847	570nm

b) Experiment 2

Time	Absorbance						
0h	0.786	0.774	0.755	0.811	0.791	0.812	570nm
2h	1.056	0.966	0.973	0.93	0.894	0.937	570nm
3h	1.181	1.106	1.093	1.043	1.005	1.049	570nm
4h	1.276	1.246	1.319	1.384	1.318	1.206	570nm
6h	1.463	1.408	1.361	1.464	1.494	1.441	570nm
24h	1.536	1.463	1.511	1.441	1.349	1.361	570nm
PBS 0h	0.763	0.732	0.746	0.761	0.835	0.750	570nm
PBS 2h	0.766	0.81	0.792	0.819	0.802	0.797	570nm
PBS 3h	0.776	0.823	0.802	0.829	0.816	0.812	570nm
PBS 4h	0.77	0.816	0.8	0.823	0.813	0.822	570nm

PBS 6h	0.767	0.814	0.785	0.81	0.806	0.816	570nm
PBS 24h	0.823	0.879	0.858	0.88	0.866	0.864	570nm

BDSQ024 activity against pre-biofilms

Table S5. *Candida* cells viability - resazurin assay (incubation time 3h). In triplicate and repeated in 2 independent experiments.

a) Experiment 1

	1	2	3	4	5	6	7	8	9	10	11	12
**A**	1.925■	1.905■	2.040■	0.711♦	0.620♦	0.685♦	0.781♦	0.654♦	0.730♦	0.700♦	0.630♦	0.737♦
**B**	0.709♦	0.636♦	0.801♦	0.726♦	0.636♦	0.74♦	0.846♦	0.858♦	0.827♦	1.74♦	1.665♦	1.814♦
**C**	2.026♦	1.973♦	2.086♦	0.688•	0.732•	0.721•						

b) Experiment 2

	1	2	3	4	5	6	7	8	9	10	11	12
**A**	1.896■	1.602■	1.911■	0.765♦	0.771♦	0.785♦	0.788♦	0.793♦	0.802♦	0.826♦	0.776♦	0.866♦
**B**	0.826♦	0.789♦	0.826♦	0.782♦	0.791♦	0.776♦	0.899♦	0.909♦	0.896♦	1.822♦	1.774♦	1.929♦
**C**	2.184♦	2.034♦	2.073♦	0.754•	0.764•	0.715•						

■ control, ♦BDSQ024: 256-2 mg/L, •Background (vehicle).

BDSQ024 activity against existing biofilms

Table S6. *Candida* cells viability - resazurin assay (incubation time 3h). In triplicate and repeated in 2 independent experiments.

a) Experiment 1

	1	2	3	4	5	6	7	8	9	10	11	12
**A**	1.038■	1.033■	1.093■	0.701♦	0.689♦	0.678♦	0.688♦	0.693♦	0.686♦	0.685♦	0.678♦	0.667♦
**B**	0.707♦	0.688♦	0.737♦	0.746♦	0.728♦	0.708♦	0.737♦	0.772♦	0.699♦	0.892♦	1.179♦	1.082♦
**C**	1.440♦	1.280♦	1.315♦	1.045♦	1.582♦	1.008♦	0.708•	0.688•	0.690•			

b) Experiment 2

	1	2	3	4	5	6	7	8	9	10	11	12
**A**	1.006■	1.053■	1.201■	0.71♦	0.736♦	0.690♦	0.734♦	0.693♦	0.716♦	0.716♦	0.740♦	0.726♦
**B**	0.731♦	0.710♦	0.719♦	0.718♦	0.702♦	0.727♦	0.862♦	0.894♦	0.882♦	1.103♦	1.250♦	1.158♦
**C**	1.451♦	1.282♦	1.422♦	1.26♦	1.605♦	1.389♦	0.711•	0.717•	0.722•			

■ control, ♦BDSQ024: 256-2 mg/L, •Background (vehicle).

References

1. Emilio José Juárez-Pérez, C.V., Francesc Teixidor, and Rosario Núñez. Polyanionic carbosilane and carbosiloxane metallodendrimers based on cobaltabisdicarbollide derivates. 2009, *Organometallics 28*, 10, doi:10.1021/om9005643.
